# A novel *KRAS* gene mutation report in sporadic colorectal cancer, from Northwest of Iran

**DOI:** 10.1002/ccr3.779

**Published:** 2017-02-09

**Authors:** Roya Dolatkhah, Mohammad Hossein Somi, Iraj Asvadi Kermani, Faris Farassati, Saeed Dastgiri

**Affiliations:** ^1^ Hematology and Oncology Research CenterTabriz University of Medical SciencesTabrizIran; ^2^ Liver and Gastrointestinal Diseases Research CenterTabriz University of Medical SciencesTabrizIran; ^3^ The University of Kansas Medical School‐Molecular Medicine LaboratoryKansas CityKansasUSA; ^4^ Tabriz Health Services Research CenterTabriz University of Medical SciencesTabrizIran

**Keywords:** Colorectal cancer, *KRAS*, mutation, sequencing

## Abstract

While the role of *KRAS* gene mutations has been widely accepted for predicting responses to anti‐EGFR therapy in patients with colorectal cancer, although this study was based on observation of a single case it gives hope that some *KRAS* gene mutation may have favorable prognosis. More studies are required on patients with similar mutation to validate this finding.

## Introduction

The pathogenesis of colorectal cancer (CRC) is complex and diverse, with different molecular pathways, and in fact leading to different phenotypes 1. Mutations in proto‐oncogenes *KRAS* and *BRAF* are among the key findings of colorectal cancer. *KRAS* mutations are present in 17–25% of all cancers and in more than 35–45% of colorectal cancer cases. The majority of *KRAS* mutations occur in codons 12 and 13 of exon 2 (more than 90%), with rare mutations at codons 61 and 146. For *BRAF* gene, more than 80% of the mutations are in the hotspot of exon 15 resulting in the *V600E* mutation in codon 600 2. Various frequencies and types of *KRAS* and *BRAF* mutations in different populations may be attributable to the effect of environmental factors, lifestyle, food habits as well as genetic factors 3, 4, 5.

Although the role of *KRAS* and *BRAF* mutations has been widely accepted for predicting responses to anti‐EGFR monoclonal antibody (mAb) treatments, particularly in patients with advanced CRCs, there is no consensus on the role of *KRAS* mutations in the prognosis and survival of patients with CRCs. While the negative prognostic role of some specific *KRAS* gene mutations in patients with colorectal cancer has been reported from a few studies, significantly shorter overall survival (OS) was observed for CRCs harboring any types of *KRAS* mutations 6, 7.

Screening of CRCs who are candidates for anti‐*EGFR*‐targeted cancer therapy protocols by detecting mutations in *KRAS* and *BRAF* genes is now used worldwide for patient selection in many oncology centers. Mutation detection of *KRAS* and *BRAF* genes, with high‐sensitivity methods, will increase the possibility of choosing the correct individual therapy and reduce the risk of metastasis in low‐stage diseases.

Although recently next‐generation sequencing was designated as a more superior technique in terms of sensitivity and specificity, Sanger sequencing has still 100% specificity and acceptable sensitivity. In other term, Sanger sequencing has a better defined track records and is most cost‐effective for routine *KRAS* and *BRAF* mutation analysis 8. Despite high cost of molecular techniques, it is now clear that routine molecular approaches of CRC are cost‐saving 9, 10.

## Case Presentation

We report a novel *KRAS* gene mutation, which was discovered while studying the prevalence and pattern of commonly involved mutations of *KRAS* and *BRAF* genes in northwest of Iran, Azerbaijan Province. The patient was a 46‐year‐old man, by Turkish‐Azeri ethnic. He was referred to our center by a history of abdominal pain and rectal bleeding having for more than 2 months. In his recent colonoscopy, he had a rectal mass. Biopsy sampling was performed during colonoscopy, and all samples were evaluated and subjected to histological diagnosis by expert pathologist. The histological analysis showed a well‐differentiated adenocarcinoma grade I, and surgery was then performed for him. Colorectal cancer (CRC) was eventually confirmed for him as stage III with no metastasis.

This patient had a history of alcohol consumption in moderate amount and never smoked. He did not have any history of chronic diseases and any other drugs or supplement intake. Of his lifestyle factors during last 6 months, vegetables and fruits consumption was one meal/day, red meat one meal/day, white bread three meals/day, and for other carbohydrate, he ate rice at least one meal/day. He drank at least three cups of black tea every day. His body weight was 100 kg, his height was 1.85 m, and his body mass index (BMI) was 29.2. He ate breakfast every day, and his circadian sleep program was in normal range (6–8 h/day). His work status was full‐time working (6–8 h/day). He did not have a regular sport program or a particular activity. He was satisfied from his life and income, married, a private house, and high school education. According to our classification of socioeconomic status (SES), he was in a very good SES class.

Chemoradiotherapy was given to him, for 6 months. The triplet chemotherapy protocol was based on the Hematology and Oncology Center protocols, including FOLFOX (oxaliplatin, 5‐FU, leucovorin). The patient has been followed up for 2 years after diagnosis. He had a good response to treatment, and he is still alive.

## Molecular Test Methods

Fresh tissues including neoplastic cells and noncancerous (tumor‐free) tissue samples from the rectal area of patient were obtained using biopsy during colonoscopy (tissue size, 2–4 mm). These biopsy samples were sent to a molecular laboratory under standard conditions and were coded and stored at −80°C.

To perform molecular tests, genomic DNA was extracted from cancer and noncancerous frozen tissue samples according to DNA Extraction Kit Guidelines (Cinnagen Company). It was taken as a template in PCR using specific *KRAS* and *BRAF* primers encompassing *KRAS* exon 2 (codons 12, 13) and *BRAF* exon 15 (codon 600) 11. PCR was conducted in 50 *μ*L final volume containing 2.5 *μ*L PCR buffer, 1 *μ*L MgCl2, 1 *μ*L from each primer, 6–7 *μ*L genomic DNA, 0.5 *μ*L dNTPs, 0.18 *μ*L of BioTaq polymerase (Cinagene), and 17 *μ*L PCR‐grade water. *KRAS* and *BRAF* were individually amplified using 2720 Thermal Cycler (Applied Biosystems, Foster City, CA).

Sanger sequencing was performed after purification of PCR products with PCR Product Purification Kit (MBST, Tehran, Iran) as per manufacturer's protocol. The reverse primers of PCR amplification of *KRAS* and *BRAF* genes were used for sequencing, using Applied Biosystem Genetic Analyzer (ABI 4‐capillary 3130 Genetic Analyzer). To ensure the accuracy of sequence results, all the molecular tests and the Sanger Sequencing analysis were performed twice for this sample.

Primary samples from tumor and noncancerous tissues were analyzed for *KRAS* and *BRAF* mutations status. PCR products were analyzed using Sanger sequencing, and their sequences were investigated using DNAMAN and Finch TV software to identify mutations in the mutant and wild‐type *KRAS* and *BRAF* genes. Codon number of the mutant gene and its changed amino acid sequence was determined by referring to NCBI/BLAST website.

Sequencing results for noncancerous tissue's extracted DNA showed the wild‐type sequence of *KRAS* gene. Moreover, mutation was not detected in the amplified exon of *BRAF* in both tumor and noncancerous tissues of patient. The status and type of mutation in *KRAS* was detected in patient included in the study, and the change in nucleotide sequence and corresponding amino acid sequence was as follows:

Sample no C104: GGA>GTA, G10V (Gly10Val), (c.29 G→T).

**Figure 1:**
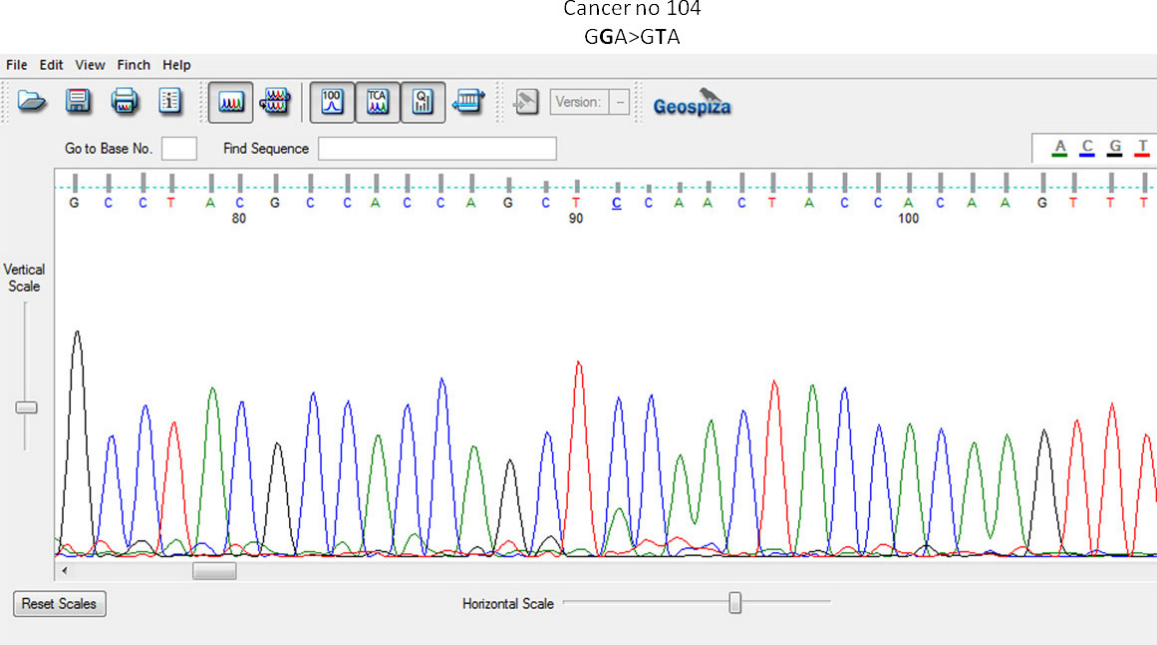
Electropherogram of KRAS gene mutation (reverse direction): GGA>GTA (G→T), codon 10 of exon 2.

**Figure 2:**
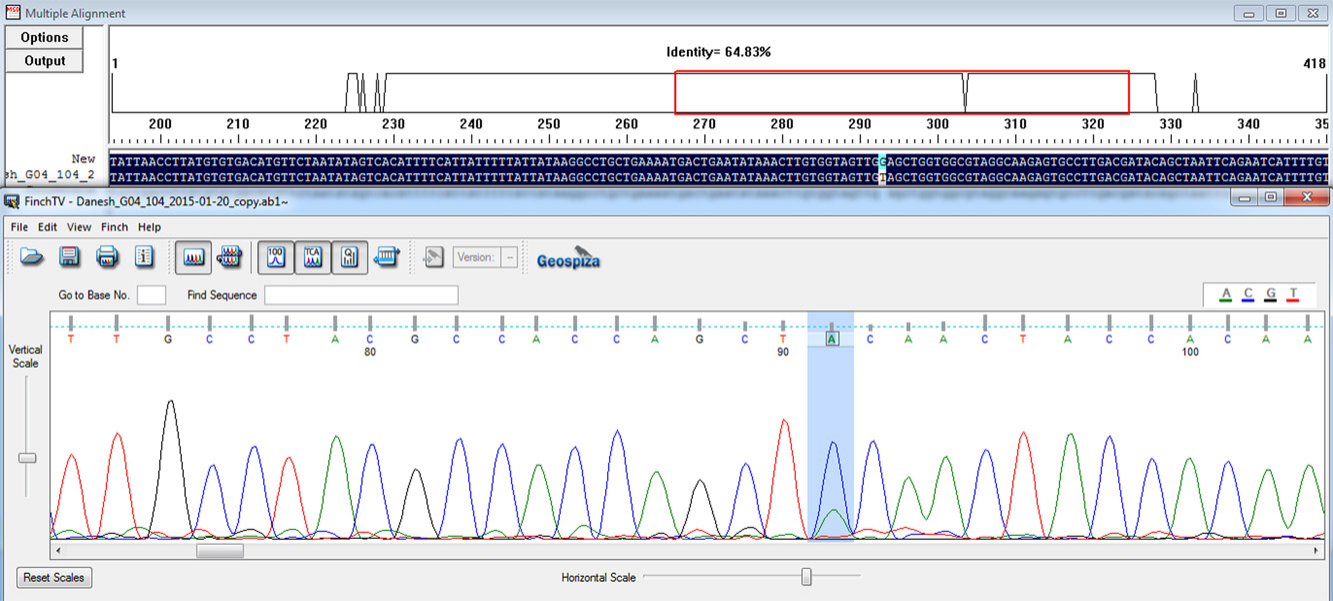
Multiple alignment and Sanger sequencing electropherogram of KRAS codon 10 mutation (reverse direction): GGA>GTA(G→T), G10V (Gly10Val).

Representative electropherograms of *KRAS* mutant are shown in Figures 1 and 2.

This is a transversion of nucleotide guanine (G) to thymine (T), in codon 10 of exon 2 of *KRAS* gene. To our knowledge, this mutation was not reported before in any database.

## Discussion

Although there is no census on the clinical significance of *KRAS* mutation in the prognosis and survival of patients with colorectal cancer, a few studies reported different outcome and overall survival in patients harboring different types of *KRAS* mutation even among commonly involved mutations in CRCs 12; however, shorter OS was observed for CRCs with any *KRAS* mutation types 6. Besides, a recent study by Fiala et al. showed that specific *KRAS* mutations including G12V/A are associated with poor outcome compared to patients with other *KRAS* mutation type. They suggested that these mutation types may confer more aggressive phenotype due to the functional differences associated with different amino acid substitutions, and then resulting protein from these mutation types. Our reported novel *KRAS* mutation G10V had the same amino acid change, but the prognosis of the patient was not as poor as their report and he had a good response to treatment 6, 12. Further experimental and cohort studies about any association between different *KRAS* mutation types and biological behaviors are needed to substantiate this theory.

This novel mutation was found to be somatic, because noncancerous tissue, as control sample, showed the wild‐type sequence analysis result. Environmental factors, food habits as well as genetic/racial/ethnic factors are believed to be responsible for discovery of this novel *KRAS* gene mutation in patient with CRC in Iran. Mutation detection methods in CRC with high sensitivity will increase the possibility of detecting new mutations in main molecular pathways of colorectal cancer. Further comprehensive molecular research studies on a large number of CRCs are needed to assess all types of mutations in these patients. This has enabled us to better characterize tumors individually and classify them according to certain molecular or genetic features.

## Ethics

The patient enrolled in the study provided written informed consent for the use of tumor and normal tissue samples for this research.

## Authorship

RD: designed the study, carried out the study, and prepared the manuscript. MHS, IAK, and FF: conceived of the study and edited the manuscript. SD: designed the study, carried out the study, and edited the manuscript.

## Conflict of Interest

None declared.
